# DEER Analysis of GPCR Conformational Heterogeneity

**DOI:** 10.3390/biom11060778

**Published:** 2021-05-22

**Authors:** Matthias Elgeti, Wayne L. Hubbell

**Affiliations:** Jules Stein Eye Institute and Department of Chemistry and Biochemistry, University of California, Los Angeles, CA 90095, USA

**Keywords:** G protein-coupled receptor, GPCR, 7TM receptor, G protein, arrestin, structure, function, structural plasticity, electron paramagnetic resonance, EPR, DEER, pELDOR

## Abstract

G protein-coupled receptors (GPCRs) represent a large class of transmembrane helical proteins which are involved in numerous physiological signaling pathways and therefore represent crucial pharmacological targets. GPCR function and the action of therapeutic molecules are defined by only a few parameters, including receptor basal activity, ligand affinity, intrinsic efficacy and signal bias. These parameters are encoded in characteristic receptor conformations existing in equilibrium and their populations, which are thus of paramount interest for the understanding of receptor (mal-)functions and rational design of improved therapeutics. To this end, the combination of site-directed spin labeling and EPR spectroscopy, in particular double electron–electron resonance (DEER), is exceedingly valuable as it has access to sub-Angstrom spatial resolution and provides a detailed picture of the number and populations of conformations in equilibrium. This review gives an overview of existing DEER studies on GPCRs with a focus on the delineation of structure/function frameworks, highlighting recent developments in data analysis and visualization. We introduce “conformational efficacy” as a parameter to describe ligand-specific shifts in the conformational equilibrium, taking into account the loose coupling between receptor segments observed for different GPCRs using DEER.

## 1. Introduction

**Figure 1:**
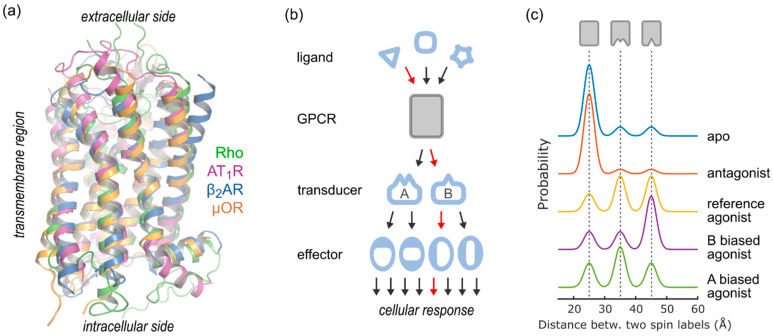
GPCR function seen from the DEER vantage point. (**a**) Structural homology among GPCRs of various functions. Receptor-specific ligands bind from the extracellular side, which therefore shows high structural diversity between different receptors. The transmembrane core exhibits many conserved interaction networks, suggesting a common activation mechanism. On the intracellular side, a limited set of transducer proteins (such as G protein, arrestin and GRK) bind to specific receptor conformations. (**b**) The GPCR “allosteric microprocessor”. Binding of an extracellular ligand is translated into ligand- and receptor-specific affinities and catalytic activities towards transducer proteins. Activated transducer proteins interact with different effectors and thereby elicit a ligand-specific cellular response. (**c**) Conformational selection from the DEER perspective. Different ligand classes (such as antagonists, balanced/reference and biased agonists) stabilize distinct GPCR conformations, leading to specific efficacies towards transducers. The different conformations and their populations are encoded as distance distributions derived from DEER. Note that all conformations are present even in the absence of ligand.

G protein-coupled receptors (GPCRs), also known as seven-transmembrane (7TM) receptors, represent the largest class of transmembrane proteins in the human genome (>800 members, [1]). Despite their homologous 7TM fold (Figure 1a), different GPCRs may bind to a large variety of different ligands, and each ligand/GPCR pair exhibits characteristic pharmacological properties such as affinity, efficacy, potency and signal bias. On the intracellular side, GPCRs couple to a relatively small set of transducer proteins such as G proteins, GPCR kinases (GRKs) and arrestins, which, once activated, bind and modulate the activity of downstream effector proteins and thus signaling pathways (Figure 1b). Hence, GPCRs function as promiscuous and yet highly specific signaling proteins (“allosteric microprocessor” [2]) channeling the binding events of distinct ligands towards different and specific physiological outcomes. This finding has been conceptualized in terms of a conformational selection model: a manifold of distinct receptor conformations coexist in equilibrium, each exhibiting differences in the orientation of structural elements, which range from individual amino acids side chains to secondary and tertiary structures. These differences translate into distinct affinities and catalytic activities of each conformation towards intracellular transducer proteins. By binding and stabilizing specific receptor conformations, ligands modulate transducer interactions and trigger a characteristic cellular signaling response (Figure 1b). In this simple framework, basal activity is achieved by residual amounts of an active conformation [3]. Biased signaling (also known as functional selectivity) is the ability of ligands to tune signaling towards a specific transducer protein, and may be understood as an equilibrium shift between active conformations of distinct affinity/catalytic activity towards a specific transducer. Traditionally, a ligand’s signal bias is quantified by comparing the cellular signaling responses to that of a “balanced” reference, such as the endogenous ligand [4]. From a biochemical standpoint, ligand binding and transducer coupling to a GPCR may be treated equally; each binding event stabilizes a specific GPCR conformational state, thereby modulating the affinity towards the other (ternary complex [5,6,7]).

In order to characterize ligand action, for example during drug design, the structural differences of functionally distinct receptor conformations and their equilibrium populations need to be determined. These characteristics may then be directly related to respective ligand parameters such as affinity, bias and efficacy (the propensity of a ligand to elicit a physiological response). Mapping the complex conformational landscape of GPCRs is a challenge for methods such as X-ray diffraction, wherein the receptor is typically removed from its native bilayer environment by solubilization in detergents and crystallized in a lattice where intermolecular forces stabilize a unique conformation. Stabilizing mutations or crystallization “helpers” are often required to facilitate crystallization [8,9,10]. Likewise, NMR methods are challenged by the slow rotational diffusion and conformational exchange of large receptor molecules, particularly in the membrane bilayer which may be essential in order to observe the functional conformational equilibrium [11,12]. Site-specific information using FRET may overcome some of the limitations of crystallography and NMR but involves large hydrophobic labels which may disrupt (local) structure and dynamics [13,14]. In addition, interpreting FRET data on a heterogeneous population in conformational exchange can be a formidable challenge. 

Site-directed spin labeling (SDSL-) electron paramagnetic resonance (EPR) spectroscopy overcomes the above-mentioned limitations of other methods and therefore provides an important complementary vantage point. SDSL-EPR is applicable to membrane proteins of any size, in detergent or lipid bilayers and can easily map ordered and disordered domains using minimally perturbing labels and small amounts of protein (picomoles). Although SDSL, like all site-specific labeling methods, provides a sparse sampling of the overall structure, this is often sufficient to characterize conformations given a starting model provided by crystallography or electron microscopy (EM).

EPR utilizes energetic transitions of unpaired electrons in an external magnetic field, which manifests in characteristic microwave absorption or emission lines. Apart from the analysis of paramagnetic metal ion centers, the use of EPR for the characterization of proteins depends on the introduction of stable radicals using SDSL. Generally, for this purpose, amino acid side chains are derivatized using a variety of different spin labeling reagents. The resulting continuous-wave (CW) EPR line shapes may be theoretically understood [15] and interpreted in terms of spin label motion in the range of 1 ps to 100 ns, which is ideally suited to monitor side chain and backbone dynamics [16,17,18]. The CW method of Saturation Transfer EPR covers the range of 100 ns to msec, well suited to monitor conformational movements [19,20]. Real-time resolution of milliseconds to seconds and beyond is possible with time-resolved CW [21]. The pulsed EPR methods of saturation recovery and electron–electron double resonance (ELDOR) provide complementary information in the µs time domain, which represents a challenging time domain for NMR [22].

SDSL-EPR applications also include a toolbox of structural methods which may be employed to characterize accessibility to paramagnetic solutes [23], in order to measure short distances in the range of 10–25 Å [24] and more [25]. The focus of this review lies on double electron–electron resonance (DEER, also known as pulsed electron–electron double resonance, pELDOR), which resolves distances between a pair of spin labels in the range of 15 to ~80 Å with sub-Angstrom resolution [26,27]. Protein structure and conformational heterogeneity is uniquely represented by analytical distance distributions, as opposed to average distances, providing a detailed picture of conformations and their populations under the chosen experimental conditions (Figure 1c). Therefore, the DEER method has proven particularly useful for the study of large, conformationally heterogeneous proteins such as GPCRs and their complexes with transducer proteins.

In the following sections we will review the conformational changes of individual GPCR segments (such as helices and loops) as reported by DEER. Table 1 summarizes all reported studies on GPCRs utilizing DEER at the time of this writing. Thereafter, we will describe an approach to globally analyze DEER data obtained for multiple label pairs (Section 2.2) and its visualization via trilateration (Section 2.3). Technical details of spin labels and the spin labeling methodology will be discussed in Section 3.

## 2. DEER Analysis of GPCR Structure and Conformational Equilibria

### 2.1. Structural Changes during GPCR Activation

#### 2.1.1. Transmembrane Helix 6

A hallmark conformational change of GPCR activation is a substantial outward movement of the intracellular segment of TM6 (helix tilt), which was first identified and structurally mapped for visual rhodopsin in detergent micelles using EPR spectroscopy [42] and DEER [28]. In accordance with 2D-EM, UV–vis, FTIR and NMR spectroscopic data, rhodopsin also forms a metastable activation intermediate [43,44,45,46], for which an intermediate TM6 position has been assessed using DEER [29]. More recently, DEER studies on other GPCRs have established a corresponding TM6 intermediate [35,38], potentially involving a counterclockwise rotation of TM6, and substantiating the existence of a common structural intermediate of GPCR activation. Importantly, studies on rhodopsin have revealed that the population of intermediate conformation in equilibrium is uniquely dependent on the membrane environment [47,48,49].

Different GPCRs exhibit distinct amplitudes of the activating TM6 tilt, which have been reported on the basis of DEER and other experimental and in silico methods [28,35,38,50,51]. These findings instigated a discussion about the different outward tilts and their functional relevance for signaling. The prevailing idea is that larger TM6 tilts, which result in a larger intracellular cavity, facilitate binding of bulkier transducer-binding epitopes than smaller TM6 tilts. Receptors capable of larger TM6 tilts are thus able to sample a wider conformational landscape with additional conformations corresponding to diverse functions. This concept was initially proposed on the basis of comparative MD simulations on β2AR and rhodopsin in order to explain G_i_ vs. G_s_ functional selectivity [51]. More recently, this idea has been corroborated by a combination of DEER and MD simulations showing that G_i_ and G_s_ stabilize distinct receptor conformations [30]. These results suggest that GPCRs and their ligands modulate signaling by a specific equilibrium position between several active conformations, in particular distinct TM6 conformations which are identifiable by DEER. An earlier study examining rhodopsin/β2AR chimeric proteins in which the TM5/TM6 connecting loop (ICL3) of rhodopsin was replaced by its much longer β2AR counterpart highlighted the importance of ICL3 length for the extent of TM6 tilt and thus G_i_ vs. G_s_ signaling [52].

**Figure 2:**
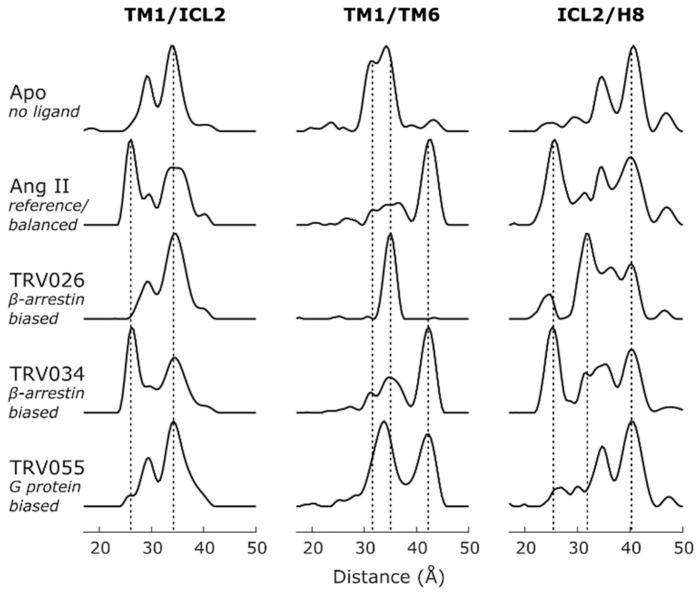
GPCR conformational heterogeneity. Distance distributions between spin labels attached to the indicated receptor segments reveal conformational heterogeneity of AT1R, which is differentially modulated by ligands of distinct function (in italics). Ang II is the endogenous, balanced agonist, while TRV026/034 and TRV055 represent β-arrestin and G protein-biased ligands, respectively. Dotted lines indicate main conformations. For this study, the cytoplasmic ends of AT1R TMs were spin labeled using IDSL (cf. [38] for the full dataset).

The population of a receptor with TM6 in an outward position has been directly correlated with receptor activity towards G protein or arrestin, which can be assayed in vitro via GTPase activity [13,53] or arrestin recruitment [6,54], respectively. Thus, antagonists, inverse, partial and full agonists can be identified by the fraction of population with TM6 in outward tilted conformation (such as TM1/TM6 distance distributions, Figure 2), meaning that DEER provides a direct measure of ligand efficacy at the level of protein conformation [55,56]. Furthermore, basal activity of GPCRs may be assessed using DEER by determining the amount of TM6 outward tilt in the absence of any ligand [3]. However, the current understanding is that a TM6 outward tilt alone is not sufficient for the determination of ligand bias towards G protein or arrestin; in this respect, TM6 tilt only represents a mandatory, not a sufficient criterion and other GPCR segments need to be characterized (see below). Also noteworthy, GPCRs in different environments, such as different detergents or membrane compositions, may adopt very different equilibrium positions of TM6 in inactive, intermediate or active conformations. Again, visual rhodopsin represents a prototypical example: in certain detergents (such as dodecyl maltoside), rhodopsin shows a quantitative shift from the inactive to the active, tilted TM6 position upon light-induced conversion of the inverse agonist to a full agonist [57]; instead, in a POPC/POPS membrane environment (nanodiscs), several distinguishable TM6 positions are populated [29]. The strong dependence of TM6 tilt on environment is likely a general characteristic of GPCRs, which specifically and non-specifically interact with molecules of the micelle or bilayer [58,59].

It is important to note that a comprehensive DEER study on the type 1 angiotensin II receptor (AT1R) resolved small, ligand-specific differences in the distance distributions of TM6, particularly with respect to peak position and shape of certain TM6 distance peaks (see Ang II vs. TRV034 vs. TRV055 in Figure 2 and [38]). This finding is somewhat reminiscent of crystallographic studies on β2AR, which reported slightly different TM6 positions stabilized in complex with the G protein G_s_ or the G_s_ mimetic nanobody (Nb80) [60,61]. The conformational landscape of TM6 in the active state appears to be rather shallow and “rugged” [62], allowing for small, ligand- and transducer-specific adjustments, which can be resolved by DEER. It remains elusive how far the small deviations from the three main conformations (inactive, intermediate and active TM6 position) affect the affinity and efficacy of ligands or transducers and thus modulate signaling. To this end, DEER will provide an important tool in future GPCR studies.

#### 2.1.2. Transmembrane Helix 5

Similar to TM6, the extracellular segment of TM5 is in direct contact with many GPCR agonists, while the intracellular end is rendering the transducer-binding pocket formed upon activation [50]. Additionally, TM5 sequences contain some of the highest conserved amino acids in GPCRs, and among those is a central proline residue (5.50, Ballesteros–Weinstein numbering, in which the first number denotes the helix in which the residue is situated and the second number indicates the relative position to the most conserved residues, which is assigned 50) which is part of the ”connector region” of GPCR activation [63] and disrupts the regular helical structure thereby introducing conformational flexibility; and Y5.58 (part of the conserved Yx_7_K/R motif) which represents an important “microswitch” of the active conformation, and mediator of signal transfer to the G protein [64]. Interestingly, most GPCR crystal structures indicate only minor structural variation in TM5 conformation. This is in accordance with DEER results on visual rhodopsin which reported only a minute inward movement of TM5 upon activation (~1Å). However, more recently, the comprehensive DEER investigation from our laboratory revealed a highly dynamic TM5 at least in AT1R [38]. In this study, complementary MD simulations were performed supporting the in vitro finding and indicating that the cytoplasmic end of TM5 frequently unravels while exploring a wide range of positions and conformations. Two possible explanations for these differences are conceivable: on the one hand, differences in the micellar or lipidic environment of the two studies may lead to specific effects on TM5 conformational space. Specific TM5/lipid interactions which are altered during receptor activation have been reported for rhodopsin on the basis of FTIR results [64,65,66]. On the other hand, TM5 conformational dynamics may be receptor specific and of functional relevance, e.g., for coupling to specific ligands or transducer proteins. Clearly, a combination of both cases is conceivable as well.

One TM5 arrangement which has been described for many GPCRs on the basis of crystal structures and verified in solution by DEER is the “helix pairing”, in which the cytoplasmic ends of TM5 and TM6 are stabilizing each other almost parallel due to conserved hydrogen bonds. This conformation is of particular importance as it represents a G α_i/o_ subtype-specific conformation and therefore may be relevant for G protein selectivity [30,51].

#### 2.1.3. Transmembrane Helix 7

EM and X-ray structures of many GPCRs have reported a small inward movement (~2 Å) as activating conformational change of TM7. The activating change of TM7 involves a pattern of changing hydrogen bonds between the highly conserved residues of the NPxxY and D/ERY motifs (Y7.53 and R3.50, respectively [67]), corroborating the notion that this conformational change is conserved among GPCRs.

In agreement, DEER studies monitoring TM7 reported an inward movement in the presence of agonists [28,29,38]; however, the observed population shift between inactive/active TM7 conformations were much less pronounced than for TM6. Even for detergent solubilized rhodopsin which otherwise converts quantitatively a small but significant population of non-active TM7 conformation remained upon light-activation [28,29]. These findings are in accordance with the notion of TM7 change succeeding TM6 tilt. For AT1R, a third conformation of TM7 has also been described, which is mostly populated in the presence of a specific β-arrestin-biased ligand (TRV026). The observed DEER distance changes are in accordance with a subtle, counterclockwise rotation of TM7, similar to the TM6 intermediate. These findings highlight the importance of TM7 for biased signaling, a notion which is compatible with ^19^F-NMR studies and more recent MD simulations [4,68,69,70]). 

#### 2.1.4. Helix 8

For most GPCRs (class A), the C-terminal end of TM7 connects via a short, structured turn to an amphipathic helix (helix 8, H8). H8 position parallel to the membrane surface is further stabilized in its inactive conformation by an aromatic π–π stacking interaction between two conserved aromatic side chains (Y7.53/F7.60, [67,71]). Importantly, H8 forms direct contacts to transducer proteins suggesting its involvement in signal transduction [31,60,72,73,74]. H8 is also common segment for post-translational modification such as prenylation of cysteine residues. These function as membrane anchors and thereby modulate conformation and dynamics of helix 8 and the attached C-terminus, which exhibits phosphorylation sites for GRK interaction [75,76].

DEER studies on H8 are so far limited to only two reports, rhodopsin and AT1R, which describe the main aspects of H8 conformational heterogeneity [28,38]. In both studies, receptor activation triggered only a minor movement of helix 8, likely orchestrated by the conformational change of the adjacent TM7, the only known connection of helix 8 to the transmembrane bundle. Furthermore, the conformational heterogeneity increased significantly with receptor activation (Figure 2). An intermediate conformation was identified which includes features of rhodopsin’s Meta I conformation such as a potential TM6 rotation [77,78], and a small changes in TM5 and TM7. Additionally, DEER data suggests an out-of-plane movement of H8 for the intermediate conformation which is likely facilitated by the absence H8 prenylation in AT1R. This finding suggests a particular importance of H8 for characteristic receptor functionality. However, further investigation of the conformational space of H8 and its dependence C-terminal post-translational modifications are required to provide a detailed picture.

#### 2.1.5. Intracellular Loop 3

Sequence analysis suggests that ICL3 may be mostly disordered in GPCRs [79], which explains the difficulties that it poses for structure determination. These challenges have been overcome by ICL3 truncation, coupling to stabilizing binding partners (transducers or nanobodies), or by inserting a soluble protein into the sequence of ICL3 as a crystallization helper [10]. However, these methods obscure native conformational states of ICL3 [80,81], which is recognized by transducer proteins [52,82] and the effect of functionally distinct ligands on ICL3 conformation would be of great interest. The lengths of ICL3 in different GPCRs differs considerably, ranging from about twenty residues to several hundred, and the high evolutionary conservation of these extended ICL3 sequences strongly suggests functional relevance [83]. In addition to specific binding motifs for cellular binding partners and phosphorylation by GRK, a longer ICL3 potentially allows for a larger amplitude of motion of the neighboring TM5 and TM6, which by itself has been reported to be crucial for certain signaling pathways (cf. section TM6, [51,52]).

So far the only DEER study including ICL3 is the rhodopsin map by Altenbach et al., which suggested an increase in flexibility upon receptor activation as determined from an increase in the width of the TM1-ICL3 distance distribution [28]. This effect likely reflects the increased conformational heterogeneity of neighboring TM5 and TM6, and fewer (tertiary) contacts between the neighboring TM5, TM6 and TM7 in the active state. Using DEER, it would be of great interest to also study the effect of ICL3 phosphorylation by GRK on its conformation and the influence on the TM conformational equilibrium [84,85,86]. Likewise, an in silico study revealed ICL3 interactions with charged lipid headgroups influencing structure and populations of signaling conformations [87].

#### 2.1.6. Intracellular Loop 2

Crystal structures of GPCRs indicate that ICL2 may exist in disordered or helical conformations and may represent an important conformational switch critically involved in G protein binding and/or activation [88,89,90]. In some instances, an interaction of ICL2 with the conserved D(E)RY motif suggests that the conformational equilibrium of ICL2 might depend on the conformation and protonation state of the transducer-binding region and thus directly modulated by extracellular ligands [91]. 

Important insights on ICL2 conformational equilibria and its functional relevance come from the comprehensive DEER study on AT1R (Figure 2, TM1/ICL2). While the labeling site 139 was originally chosen on the basis of an existing crystal structure as a stable TM4 reference site for distance measurements, it turned out that this site undergoes a large, ligand-induced conformational change. This was confirmed by investigation of a second reference site in TM1 (DEER mapping, see Section 2.3) and by complementary MD simulations which indicated that TM4 may often unravel at the cost of an extended and conformationally heterogeneous ICL2. We found that the ICL2 conformational switch is activated predominantly by β-arrestin-biased ligands (i.e., ligands which do not exhibit significant efficacy towards G protein), suggesting that this switch leads to an “occluded active” conformation unable to bind or activate G_q_ [38]. 

#### 2.1.7. GPCR/Transducer Complex

Transducer proteins have extended contacts to GPCRs by which they recognize active receptor conformations and some of which lead to conformational changes within the transducer [92]. In turn, these contacts stabilize respective receptor conformations, leading to a shift of the conformational equilibrium. DEER has been utilized in different ways to characterize GPCR/transducer complexes. DEER was successfully applied to singly spin-labeled GPCRs and transducers in order to demonstrate complex formation and as a verification of the architecture of crystallographic GPCR–transducer complexes (Table 1). Both the rhodopsin/arrestin-1 and rhodopsin in complex with heterotrimeric G protein (Gα_i_βγ) have been analyzed by DEER and good agreement with respective crystal structures has been assessed. The DEER distance distributions indicate residual structural heterogeneity, especially for the rhodopsin–arrestin-1 complex. This finding suggests that either the receptor or the transducer exist in more than one conformation, a hint at different architectures of this complex with potentially distinct function [31,93]. DEER, due to its superior applicability to large, structural heterogeneous proteins and protein complexes, represents a predestined experimental method to track down those alternative “flavors” [94].

DEER distance distributions have also been reported for doubly spin-labeled receptor in complex with (unlabeled) transducers or mimetics thereof. For rhodopsin in a lipidic environment, it was shown that binding of the cognate G protein G_i_ lead to a substantial shift towards the active conformation in both TM6 and TM7, leading to depopulation of unbound rhodopsin [29]. However, while TM6 shift was quantitative at chosen receptor and G protein concentrations, for TM7, a small but substantial fraction of inactive conformation remained. This finding corroborates that TM6 and TM7 conformation are not strictly coupled to one another. Further, binding of G protein to TM7/H8 of the receptor, as indicated by crystal structures, is not mandatory for G protein binding but might play an additional, downstream role, e.g., for activation of the G protein α-subunit (nucleotide exchange) or G protein release. In contrast, binding of a G_q_ mimetic nanobody to AT1R stabilized all transmembrane helices including TM7 in the active conformation [38]. This finding may indicate potential differences in G_i_ versus G_q_ binding or, more likely, the specific nature of the nanobody which was matured to exhibit highest affinity and stabilize a structurally homogeneous receptor population for crystallization [39].

#### 2.1.8. GPCR Dimers

Homo- and hetero-oligomerization of GPCRs has been shown to occur for several GPCRs [95], and its functional relevance for signaling and signal bias is currently under scrutiny [14,96]. In principle, dimerization can also be assessed using DEER, using the intensity of the DEER signal modulation as measure of the number of coupled spins per nano-object [97]. However, GPCR oligomerization shows strong concentration dependency and cell-surface expression levels of most GPCRs are below the concentrations commonly used in DEER. These challenges may be overcome by complementing DEER with other techniques, such as fluorescence spectroscopy or simulations [36]: While DEER provides superior resolution and an ensemble picture, application of (single-molecule) FRET is suitable at very low concentrations provided by native GPCR expression levels [14]. Instead, simulations may evaluate different dimer assemblies and their agreement with site-directed labeling (DEER or FRET) results, and allow extrapolation towards all-atom structures of the oligomer [98]. Conversely, in the case of rhodopsin, receptor molecules are tightly packed in the disc membrane of rod outer segment (25,000 molecules/μm^2^) complicating DEER analysis by imposing a strong background signal [34]. Here, nanodiscs and other lipidic environments such as polystyrene-co-maleic acid lipid particles (SMALP) may provide a possible solution for the characterization of GPCR oligomers by DEER [32,99]. These platforms compartmentalize receptor molecules into independent lipidic nano-particles, thereby providing tight control of GPCR to lipid ratios even at the micromolar concentrations typically used for DEER.

### 2.2. Non-Negative Matrix Factorization

Intriguingly, even under conditions of saturating ligand concentration, the multimodal distance distributions obtained from DEER indicate the coexistence of several conformational states. As demonstrated in several DEER studies and exemplified in Figure 2, the populations of conformational states appear shifted in the presence of distinct ligands. This suggests that the underlying conformational states share a common equilibrium. The results also reveal uneven population shifts for different spin label pairs in response to changes in ligand condition. This may indicate that specific label positions disturb the local protein structure, leading to an altered conformational equilibrium, which can be ruled out, e.g., by evaluating ligand binding of each spin-labeled receptor [38]. Instead, the uneven population shifts reflect an increase in conformational heterogeneity, with individual receptor segments only loosely coupled in the active state. The concept of “loose allostery”, consistent with the considerable increase in conformational entropy occurring with rhodopsin activation [46,48], is gaining traction in GPCR research [63,100]. From the DEER perspective, multimodal distance distributions and uneven conformational responses complicate the assignment of specific distance peaks to global conformational states. 

A possible solution is to investigate all spin label pairs under a variety of different conditions, such as different ligand conditions, and correlate the observed distance peaks with respect to their populations. Common ways to combine different observables include principal component analysis (PCA), singular value decomposition (SVD) or independent component analysis (ICA), all of which summarize a given dataset in a reduced number of components *C* and their loadings *P*. Due to the non-negative nature of DEER distance data, non-negative matrix factorization (NNMF) can be utilized in order to identify conformations and equilibrium positions [38,101]. NNMF factorizes a given (non-negative) data matrix *M* (each column *M*_i_ containing the area normalized distance distributions from all spin label pairs recorded under identical conditions) into two smaller (non-negative) matrices *C* and *P* so that ${\left\|M-C\times P\right\|}^{2}$ is minimized. NNMF requires selection of a number of *n* columns and rows for the resulting matrices *C* and *P*, respectively, so that their product $M^{\prime }=C\times P$ becomes a reasonable approximation of the original data matrix *M*. For DEER, *n* reflects the number of conformations observed in the dataset *M* and may be identified by comparing *M*′ calculated for a variable number *n*. The most parsimonious model (smallest *n*) is identified by model selection methods using information criteria such as the Akaike information criterion corrected (AICc) or the Bayesian information criterion (BIC) [102]. Notably, it is important to not blindly accept the results from model selection tools but to rather use them as guidance for the selection of an appropriate model. 

**Figure 3:**
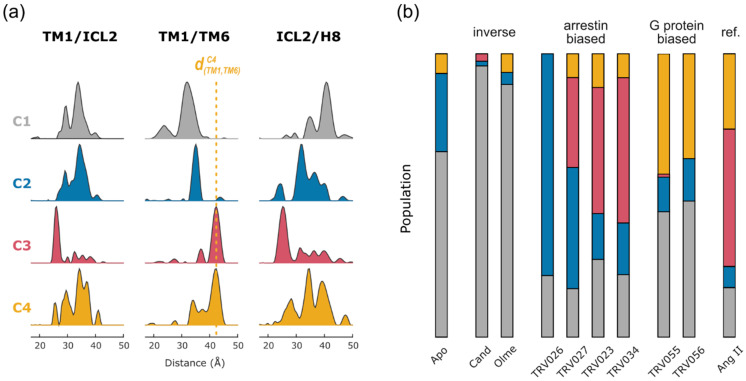
NNMF analysis. (**a**) NNMF analysis of AT1R DEER data reveals four major conformational states *C1–C4* (columns of matrix *C*). Residual multimodality in each conformation suggests Table 1. *C4* under the various ligand conditions. Bias of different agonists can be identified by the population of *C2*, *C3* and *C4*.

In the case of AT1R, we collected DEER data on ten spin pairs covering the cytoplasmic receptor surface under ten different ligand conditions. NNMF yielded four conformations *C* (Figure 3a), and the associated matrix *P* provided a detailed picture how these conformations are differentially populated under each ligand condition (Figure 3b). Compared to G protein-biased agonists and the endogenous reference agonist Ang II, arrestin-biased ligands did not stabilize large amounts of conformation *C4* but stabilized *C2* and *C3* instead. Accordingly, the population of *C4* showed high correlation with the ligand’s ability to trigger G protein activation [38]. These findings provide structural underpinnings of biased signaling in AT1R and possibly in GPCRs in general.

Interestingly, the distance distributions of the resulting conformations *C1*–*C4* still show considerable multimodality, at least in some spin pairs (e.g., for those involving ICL2, Figure 3a). This demonstrates that the identified four conformations represent main energy wells in the conformational landscape and that conformational substates exist reflecting local plasticity. Since DEER experiments are performed on an ensemble under equilibrium conditions, strict (energetic and structural) coupling between individual receptor segments remains to be elucidated by other means such as kinetic experiments. 

### 2.3. DEER Distance Mapping

**Figure 4:**
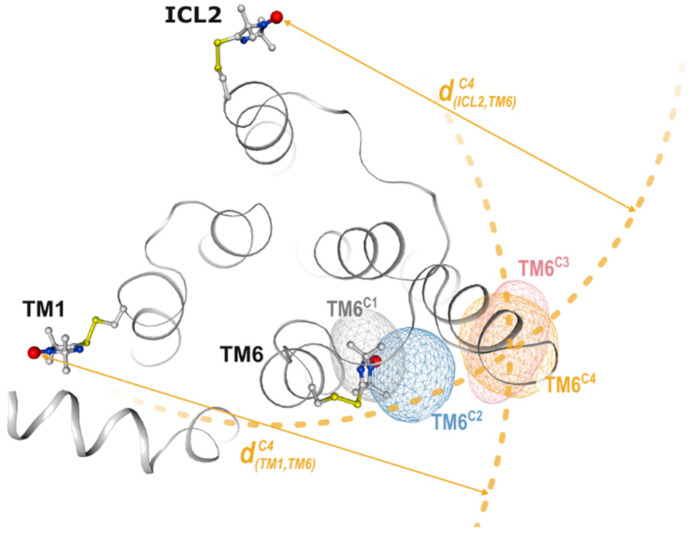
DEER mapping. Cytoplasmic surface of AT1R with spin label side chains modeled indicating the reference sites, TM1 and ICL2, and the monitor site TM6. Shown spin densities were calculated from main NNMF distances via 2D trilateration. For TM6^C4^, the used distances d^C4^ and intersecting spheres (dotted lines) are shown for visualization. The underlying structure was derived from MD simulations of antagonist bound AT1R [38].

Distance mapping using DEER distance distributions was introduced for GPCRs by Altenbach et al. using the rhodopsin model system [28]. In this study, the characteristic TM6 outward tilt, the fingerprint of GPCR activation, was first directly detected. The mapping approach utilizes the distance constraints from multiple spin label pairs in order to characterize conformational changes in two (or three) dimensions, which is helpful for the comparison with structural information from other techniques (e.g., X-ray/EM structures, Figure 4). At least two (or three) reference labeling sites need to be chosen, which are characterized by their inter-residue distances remaining the same across all conditions to be investigated. It is with respect to these reference sites that all other distances and populations are characterized. Instead, monitor sites are on the protein segments which undergo conformational changes and should be chosen within the distance range accessible to DEER (15–80 Å). Structural information from X-ray crystallography and EM, sequence alignment and published DEER studies (Table 1) should be consulted in order to identify suitable reference and monitor sites. For low-expressing proteins such as GPCRs for which conformational changes of TMs in the membrane plane are of primary interest, it is appropriate to pick all labeling sites approximately within the same plane. This simplifies the conformational landscape to two dimensions and significantly reduces the number of experiments required for mapping. 

In order to map a specific protein conformation, peaks in each distance distribution need to be assigned to this conformation. For detergent solubilized rhodopsin, the population shifts occurring with activation are quantitative making the assignment of distance peaks to inactive or active conformations straightforward. In the case of AT1R, however, DEER distributions appeared strongly multimodal, indicating more than two conformations in each ligand state (cf. Figure 2). As described above, NNMF analysis identified variable mixtures of only four main conformational states in equilibrium, each represented by a set of characteristic distance distributions and an associated population for each condition. This simplified selecting the distance peaks for each conformation to be mapped (Figure 3a).

Initial coordinates of the nitroxides of reference and monitor sites may be retrieved from crystal structures using spin label modeling software such as MMM [103], Charmm-GUI [104], Pronox [105], MtsslSuite [106] or others. After peak assignment for each conformation, the coordinates of all monitor sites undergo a global optimization to account for the differences between starting coordinates and DEER data, e.g., due to a conformational change. In the next step, for each individual spin label, the probability is calculated for all points in the 2D (or 3D) space using the optimized label coordinates of all other sites and all DEER distance distributions (Figure 4). Finally, these probabilities may be visualized as two- (or three-)dimensional densities and superimposed onto a reference structure. This way, different conformations of the same receptor segment can be directly compared giving a unique vantage point on GPCR conformational heterogeneity (Figure 4, [28,38]).

### 2.4. Conformational Efficacy

In principle, ligand affinity can be determined through DEER measurements, for example by placing a monitor spin label close to the ligand-binding site. In practice, recording titration curves using DEER is tedious and costly compared to established methods that are routinely used for this purpose [107]. Thus, in all reported DEER studies, ligands were added in excess, essentially characterizing the conformational equilibrium at the high concentration endpoints of the ligand-binding curve.

Much can be learned from the conformational equilibrium stabilized under saturating ligand conditions, especially when several functionally distinct ligands are tested (cf. Figure 2). Ligands of variable intrinsic efficacy can be easily identified by their distinct populations of inward, intermediate and outward TM6 positions [3,35]. If several spin pairs are investigated under identical conditions and, if necessary, a global analysis such as NNMF is performed, transducer-specific active conformations can be identified and structurally mapped. This has been demonstrated for AT1R and its interaction with G_q_, in which case the population of a specific conformational state (NNMF component *C4*) in the presence of different ligands was highly correlated with the ligands’ efficacy towards G_q_ signaling [6,38]. This “conformational efficacy”, namely the ligand’s propensity to stabilize a specific conformation (of a certain function), appears to be one of the critical ligand parameters and is directly accessible by DEER. In light of the uneven population shifts observed for distinct receptor segments, (global) conformational efficacy can be broken up even further into segment-specific efficacies.

Taken together, the extension of the traditional GPCR conformational selection model to include loose coupling of individual receptor segments, the role of individual conformational changes and their inter-segmental coupling are major challenges of GPCR research. DEER will be at the forefront of these studies, facilitating the development of highly efficacious and specific therapeutics with fewer adverse effects, as well as providing a deeper understanding of membrane protein function in general.

## 3. Site-Directed Spin Labeling (SDSL) and Reagents to Modify Cysteines

Introducing paramagnetic spin centers to a protein for EPR studies can be achieved in many different ways [108]. Most common is the site-specific introduction of a cysteine residue at a solvent-exposed site, followed by derivatization of the reactive sulfhydryl by a thiol-specific spin labeling reagent. Selective labeling of introduced cysteines does require replacement of any solvent-exposed and reactive native cysteines with a suitable substitute (such as serine). Such solvent-exposed native cysteines are generally not functionally important, but GPCR sequences do contain conserved buried or partially buried cysteines and functionally important cystines, the latter of which could in principle be involved in sulfhydryl-disulfide exchange equilibria [109]. 

**Figure 5:**
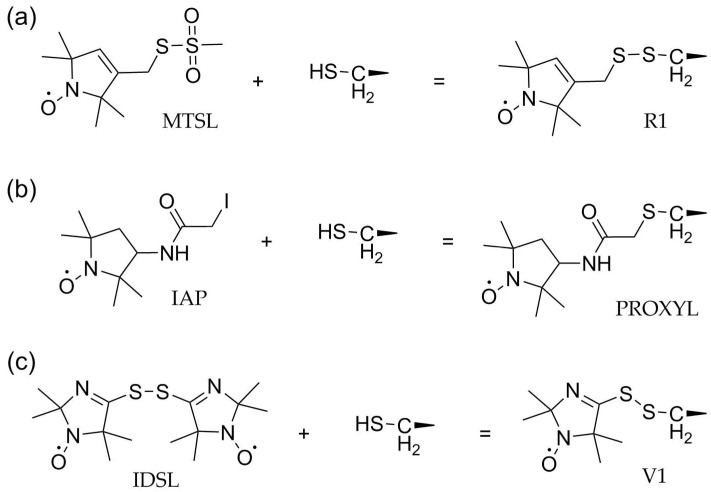
Spin labels commonly used for DEER. (**a**) MTSL: Reaction with the thiol side chain creates the disulfide-linked side chain R1. (**b**) IAP: S_N_2 reaction creates the thioether-linked PROXYL side chain. (**c**) IDSL: Sulfhydryl-disulfide exchange leads to formation of the short spin label side chain V1.

Even after removal of solvent-exposed cysteines, more solvent inaccessible residues, if present, can react to a limited extent, and such background labeling and its consequence must be evaluated in each individual case. Thus, replacing solvent accessible, non-critical cysteines with suitable non-reactive amino acids (the pseudo-wild type), and optimizing labeling conditions to minimize background labeling may require considerable experimental groundwork. These steps should be guided by CW-EPR and by determination of labeling efficiency to guarantee site-specific labeling and optimize yield. Ideally, sites for the labels should be chosen to lie at non-interacting solvent-exposed surfaces of the protein to insure the absence of structural perturbation. Such sites are typically selected based on a structural model of the protein derived from either crystallographic studies or molecular modeling. In the case of the commonly used R1 nitroxide side chain (Figure 5a), a proper non-interacting surface site is readily confirmed by the signature CW-EPR line shape that arises from the well-characterized anisotropic internal motion of R1 [17,18]. If the site selected from the model does not have the anticipated line shape, the model apparently does not represent the actual structure under the experimental conditions, and nearby sites can be tested. It is desirable that the sites selected for spin labeling be such that the conformation of the side chain (rotameric state) be insensitive to the conformational state of the protein. This latter condition provides insurance that distance changes measured by DEER reflect true spatial rearrangement of the protein with minimal contributions from rotameric changes of the labels. Again, if R1 is employed, this condition can be confirmed directly by the CW line shapes, which should not change with protein conformation. Other spin labels (discussed below) may be immobilized relative to the protein due to internal side chain interactions and the CW line shapes contain little information on dynamics or local interactions of the side chain. However, for such labels the strong internal interactions of the side chain that lead to the immobilization suggest that single rotamers are selected with little probability of rotameric changes due to changes in conformation of the protein. For strongly immobilized spin labels it is particularly important that solvent-exposed sites be selected because, unlike R1, such side chains cannot accommodate steric clashes by rotameric adjustments; rather, the protein is forced to repack. R1 has the ability to adapt to local structure with little energetic cost and is much less perturbing if buried or partially buried sites must be employed. 

In the following paragraphs, several spin labeling reagents and the nitroxide side chains resulting from their reaction with cysteine are briefly discussed. Coverage of the topic is restricted to classes of spin labels used so far in DEER studies of GPCRs.

### 3.1. Methanethiosulfonate Spin Labels (MTSL)

MTSL react rapidly and specifically with cysteine over a wide range of pH. Several nitroxide methanethiosulfonate reagents have been employed in spin labeling, but MTSL (2,2,5,5-tetramethyl-pyrroline-1-oxyl methanethiosulfonate) is by far the most commonly used to date. The reaction of MTSL with cysteine generates the disulfide-linked nitroxide side chain designated R1 (Figure 5a). The reactivity of MTSL is sufficiently high for solvent-exposed cysteines that a 1:1 stoichiometry of reagent to cysteine has been found to proceed to near completion in minutes even at micromolar concentrations [110]. This makes it possible to selectively target highly reactive exposed cysteines in the presence of less reactive buried and partially buried residues. R1 has been extensively employed for the characterization of the GPCR rhodopsin (Rho) using CW-EPR and DEER. R1 has a size and polarity similar to that of regular amino acid side chains and crystallographic studies have shown it to adopt mainly three distinct rotameric states, each of which has a similar anisotropic internal motion and hence similar CW-EPR spectrum. The well-characterized internal motion makes R1 very sensitive to local secondary and tertiary structures, and this label has been extensively used in mapping of secondary structure [111]. However, the fact that the rotamer distribution may be determined by local interactions leads to ambiguity in the distance distributions determined by DEER, since the position of the nitroxide group itself may be different in each rotamer, giving rise to increased widths of distance distributions determined by DEER. In addition to MTSL many other methanethiosulfonate nitroxide reagents and the corresponding side chains resulting from reaction with cysteine residues have been investigated [17]. Substituents on the 4-position of the nitroxide pyrroline ring reduce the rotameric space and strongly inhibit the internal motion of the side chains. For example, a crystal structure of the 4-phenyl derivative reveals a single highly ordered rotamer, while the 4-pyridyl apparently adopts a single rotamer in solution as indicated by proton enhanced relaxation spectroscopy [112] Thus, 4-substituted derivatives of R1 are expected to be valuable DEER labels for future studies of GPCRs and other proteins. The most highly restricted and localized spin label is the cross-linked side chain designated RX generated by the corresponding bis-methanethiosulfonate. However, use of this reagent for both spins requires the introduction of four cysteine residues [113].

### 3.2. Activated Alkyl Halides 

Activated alkyl halides are generally less reactive towards cysteine and less specific than methanethiosulfonates. The reaction mechanism (S_N_2) involves a back-side attack by the nucleophile (S^-^) with inversion of configuration at the carbon bonded to the halide. The stereochemical requirement apparently limits the rate of reaction for cysteines in a confined environment. For example, the iodoacetamide IAP (Figure 5b, (3-(2-Iodoacetamido)-2,2,5,5-tetramethyl-1-pyrrolidinyloxy) fails to react with the two partially exposed native cysteines in rhodopsin, both of which rapidly react with MTSL (WLH, unpublished results). However, it is also less selective than MTSL and can undergo nucleophilic reaction with the primary amine of lysine. This reaction becomes important at basic pH where the amount of unprotonated lysine becomes significant (ca. pH > 8.5). The saturated proxyl ring of IAP has an asymmetric center and the label is synthesized as a mixture of isomers. Finally, the PROXYL ring of IAP allows relatively unrestricted motions about the terminal bonds adjacent to the ring [17]. These latter two features can potentially lead to broad interspin distances determined and reduced resolution in DEER [3,35]. The sole advantage of IAP as a spin label is that the reaction with cysteine generates a thioether linkage stable to reducing agents such as TCEP and DTT, unlike the disulfide linkage of R1 which is rapidly cleaved by these reagents. This can be an advantage for proteins that require a reducing environment.

Another activated alkyl halide that has been used for myosin labeling is the iodoketone designated IKSL [114]. The unsaturated ring of IKSL eliminates some of the problems mentioned for IAP related to the PROXYL ring, and the side chain generated by the reaction with cysteine is highly immobilized, suggesting a more localized nitroxide. While IKSL may have significant advantages over IAP, it is still subject to low reactivity and specificity of alkyl halides in general.

### 3.3. Disulfides

Sufficiently reactive disulfides can also act as spin labeling reagents for cysteine via a sulfhydryl-disulfide exchange reaction. The sole example of this class that has been employed as a spin labeling reagent is the bis(2,2,5,5-tetramethyl-3-imidazoline-1-oxyl-4-il)-disulfide spin label (IDSL, also known as RSSR, Figure 5c). The reaction product with cysteine is the disulfide-linked spin label side chain designated V1 [109,115]. Extensive analysis using CW-EPR, X-ray crystallography and quantum chemical calculations revealed an intra-side chain S-N stabilizing interaction, which strongly reduces both the rotameric space and the side chain flexibility [115,116], making IDSL an attractive label for DEER. The imidazoline function renders the sulfur adjacent to the ring electrophilic, susceptible to nucleophilic attack by hydroxide ion [109] resulting in cleavage of the spin label under basic conditions (ca. pH > 7.5). The details of the mechanism may be complex, but the product is the free imidazoline thiolate anion. The cleavage reaction, if present, is evident in the CW line shape. For DEER, the slow hydrolytic reaction is of little consequence because the sample is rapidly frozen after labeling. 

The activated disulfide of V1 is also susceptible to attack by an S^−^ nucleophile from a nearby cysteine in the protein, again releasing the free imidazoline thiolate. In this sulfhydryl-disulfide exchange reaction, V1 acts as an oxidizing reagent. In our recent comprehensive investigation of the angiotensin receptor (AT1R), this reaction may have taken place, resulting in the oxidation of two nearby native cysteines to cystine, allowing the selective labeling of the exposed engineered cysteine. Both MTSL and IAP consistently and stably derivatized the native cysteines as well as the engineered cysteine giving rise to complex distance distributions in DEER. Similar behavior was recently observed in preliminary DEER data on the Y2 receptor [40].

## 4. Conclusions and Outlook

This review aimed to summarize the conformational changes occurring with activation of GPCRs; we limited our explanation to the vantage point of DEER, which is unique as it provides an accurate lengths scale and can resolve multiple conformations in equilibrium, which is the case for GPCRs. The reader is referred to excellent reviews focusing on other aspects of GPCR research [2,4,63,92,117].

In recent years, SDSL-EPR spectroscopy, and in particular DEER, has being recognized as an important technique for uncovering the structural underpinnings of GPCR function. Indeed, SDSL does not yield all-atom structural information. However, in combination with other methods, e.g., computational tools, new GPCR conformations may be identified potentially exhibiting functional relevance and therefore presenting important drug targets [30,118]. Moreover, the site-directed labeling approach provides superior resolution compared to other, global spectroscopic methods such as NMR or FTIR spectroscopy, where frequency assignment often represents a major challenge. Finally, DEER provides the important length scale to the description of conformational landscapes. This also facilitates direct comparison with structural information derived from X-ray crystallography, electron microscopy or MD simulations, all of which may provide complementary all-atom resolution.

DEER requires rapid freezing in order to extend the labels phase memory time T_2_* into the microsecond time regime and to trap the protein conformational equilibrium. Experimental evidence indicates that the conformational equilibrium at ambient temperature is effectively trapped by flash freezing [119,120,121], which is further corroborated by other distance measurement techniques (such as smFRET [13,122] and relaxation enhancement EPR [123]). To avoid low temperatures, progress is being made in label development to achieve phase memory times (T_2_*) long enough for DEER at physiologically relevant temperatures [124,125].

A further important factor to consider when interpreting DEER data in a physiological context is the in vitro environment, since detergent or membrane environments exert a strong and specific influence on the stability and conformational equilibria of GPCR conformations. However, this topic is out of the focus of this review and the reader is referred to specialized publications [126,127].

The vast conformational heterogeneity of GPCR indicated by broad DEER distance distributions poses challenges for structure determination. The modulation of conformational equilibria by ligands of diverse function also indicates the vast potential for pharmacological research in order to find selective and efficacious therapeutics. To resolve the conformational equilibrium using DEER, progress is being made in terms of data analysis including model-based fitting tools which provide the statistical rigor to a fully reproducible DEER analysis [128,129].

DEER may be used to investigate GPCR homo- and hetero-dimerization, important regulators of GPCR function and potentially involved in signal bias [14]. This will be facilitated by the commercial availability of large volume Q-band cavity resonators and other instrumentation, which allow for sample concentrations in the physiological regime. Progress towards more dilute samples and the development of reduction-resistant spin labels will also boost *in-cell* DEER investigations [130]. Another promising avenue is the application of hydrostatic pressure, which, in combination with DEER, shows great potential for exploring the conformational landscape of GPCRs by stabilizing rare conformations and elucidating the thermodynamics of ligand binding and conformational change [3,131,132].

## Figures and Tables

**Table 1:** Published DEER studies.

GPCR ^a^	Transducer	Distances ^b^	System ^c^	Label ^d^	Ref.	Note
Rho		TM1-H8	DDM pH6	MTSL	[28]	First study to directly detect conformational changes of GPCR activation
	G_i_	TM2-TM5 TM2-TM6 TM2-TM7	Nanodiscs DDM var pH	MTSL	[29]	Effect of lipidic environment on GPCR conformational equilibria
	G_i_	intermolecular	DDM	MTSL	[30]	Architecture of rhodopsin–Gi complex
	arrestin-1	intermolecular	Nanodiscs	MTSL	[31]	Crystal structure of the rhodopsin–arrestin-1 complex
		TM3-H8	Nanodiscs membranes	MTSL	[32]	Rhodopsin dimer in nanodiscs
	G_i_	TM2- TM5/TM6/TM7	DDM	MTSL	[33]	EM structure of rhodopsin–Gi complex
		TM3, H8 (native)	Native membranes	MTSL	[34]	Characterization of native rhodopsin oligomers
β2AR	Nb80	TM4-TM6	DDM/CHS	IAP	[35]	Characterization of ligand-induced equilibrium shifts and loose allosteric coupling
	Nb80	TM4-TM6	DDM/CHS	IAP	[3]	Pressure resolved DEER identifies small amounts of active receptor responsible for basal activity
NTS1		TM1–7, H8	liposomes	MTSL	[36]	Dimer mapping, DEER stitch [37]
AT1R	Nb	TM1-ICL2 TM1-TM6/7/H8 ICL2-TM5/6/7/H8 TM5-H8 TM6-H8	MNG/CHS	IDSL	[38]	Conformational signatures of GPCR-biased signaling
AT1R	EC-Nb	TM1-TM6 ICL2-TM5/H8	MNG/CHS	IDSL	[39]	Using nanobodies as highly specific GPCR ligands
Y2R		TM3-TM7	bicelles	MTSL/IDSL	[40]	Conformational changes in refolded GPCR
GCGR		TM4-TM5 TM4-TM6	MNG/CHS	IDSL	[41]	Differences in the activation mechanisms of class A and class B GPCRs

^a^ GPCR abbreviations: Rho, rhodopsin; β2AR, β_2_-adrenergic receptor; NTS1, neurotensin 1 receptor; AT1R, type 1 angiotensin II receptor; Y2R, neuropeptide Y receptor; GCGR, Glucagon receptor. ^b^ The segments between which interspin disances were measured. ^c^ DDM, dodecyl maloside; CHS, cholesteryl hemisuccinate; MNG, lauryl maltose neopentyl glycol. ^d^ MTSL, methanethiosulfonate spin label; IAP, iodoacetamido proxyl spin label; IDSL, imidazoline spin label (structures given in Figure 5).
